# Ectopic Thyroid Tissue in the Mediastinum Characterized by Histology and Functional Imaging with I-123 SPECT/CT

**DOI:** 10.1155/2017/9084207

**Published:** 2017-01-30

**Authors:** Jed Hummel, Jason Wachsmann, Kelley Carrick, Orhan K. Oz, Dana Mathews, Fangyu Peng

**Affiliations:** ^1^Department of Radiology, UT Southwestern Medical Center, Dallas, TX, USA; ^2^Department of Pathology, UT Southwestern Medical Center, Dallas, TX, USA; ^3^Advanced Imaging Research Center, UT Southwestern Medical Center, Dallas, TX, USA

## Abstract

Ectopic thyroid tissue is a rare entity and when discovered it is typically along the pathway of embryologic migration of the thyroid. We present a case of incidental finding of ectopic thyroid tissue within mediastinum in a 61-year-old female patient with a history of total thyroidectomy for thyroiditis and nodules. The patient presented to emergency room with cough and right chest pain and underwent a chest computed tomographic angiogram (CTA) to exclude pulmonary embolism as part of chest pain workup. One right paratracheal mediastinal soft tissue nodule was visualized on the images of CTA. This right paratracheal soft tissue mass was found to be ectopic benign thyroid tissue by histological analysis of the biopsied tissue samples. The function of this ectopic thyroid tissue was characterized by I-123 radioiodine uptake and single photon emission computed tomography/computed tomography (SPECT/CT) imaging. This case illustrates that ectopic thyroid tissue should be included for differential diagnosis of a hyperdense soft tissue mass located within mediastinum. I-123 SPECT/CT is useful for guiding tissue biopsy of ectopic thyroid tissue distant from orthotopic thyroid gland and functional and anatomic characterization of mediastinal ectopic thyroid tissue for surgical resection when it is medically necessary.

## 1. Introduction

Ectopic thyroid tissue is a rare entity and when discovered it is typically along the pathway of embryologic migration of the thyroid [1]. The embryologic development of the thyroid provides an anatomic roadmap for the typical locations of ectopic thyroid tissue. The thyroid gland originates from an endodermal thickening between the first and second pharyngeal arches. There is a caudal migration of the thyroid primordium from the foramen cecum of the tongue to the thyroid bed at the pretracheal neck base, typically positioned anterolateral from the second to the fourth tracheal cartilage. This pathway of descent is marked by the thyroglossal duct. The thyroid primordium passes anterior to the hyoid bone and then loops inferiorly and posteriorly to the hyoid bone. There are circumstances in which thyroid tissue may be found outside the gland due to faulty embryogenesis related to genetic factors, mechanical implantation secondary to surgical intervention or trauma, a sequestered thyroid nodule adjacent to the gland but without anatomic connection, or thyroid tissue as a component of a teratoma [1–4]. The lingual area is the most common location of ectopic thyroid tissue [3, 5, 6], and ectopic thyroid tissue is occasionally localized within mediastinum [7–13]. Most of patients with ectopic thyroid presented with symptoms of hypothyroidism, and rare cases of hyperthyroidism with histological features similar to Graves' disease have been reported [14–18]. Some patients may present with symptoms such as cough, dysphagia, dyspnea, stridor, and dysphonia related to mass effect on regional structures [16]. Ultrasound of neck was often used for detection of ectopic thyroid and radioiodine uptake and radioiodine scan could be used for functional characterization of suspected ectopic thyroid tissues [19]. Hybrid SPECT-CT imaging is a useful imaging modality for both functional and anatomic evaluation of suspected ectopic thyroid tissue, particularly those located at an unusual location distant from expected location of ectopic thyroid tissues along the thyroglossal duct [6, 20, 21]. Herein, we report a case of incident finding of an ectopic thyroid tissue mass within the mediastinum in a patient with remote history of total thyroidectomy for thyroiditis and nodules. The patient presented to emergency room with cough and chest pain and underwent CTA to exclude pulmonary embolism as part of workup of right chest pain. On CTA images, one right paratracheal nodular soft tissue mass was visualized, which was found to be ectopic thyroid tissue by histological analysis of tissue sample from endoscopic bronchial ultrasound-guided biopsy and functional imaging with I-123 SPECT/CT.

## 2. Case Presentation

**Figure 1:**
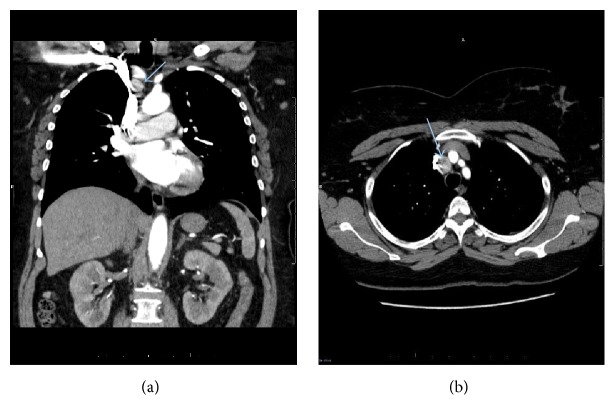
A right paratracheal mass in the upper mediastinum on the images of CTA from a patient presented with cough and right chest pain. A 61-year-old female presented to emergency room with cough and right chest pain. Chest CTA was performed to exclude pulmonary embolism as part of chest pain workup. One 2.0 × 1.7 cm right paratracheal mediastinal mass was visualized on the images of CTA, as indicated by a blue arrow on coronal (a) and axial (b) view images of CTA. This right paratracheal mass appeared hyperdense or showed mild contrast enhancement relative to other small mediastinal lymph nodes.

A 61-year-old female presented to the emergency room with cough and chest pain. A CTA of the chest was performed to exclude pulmonary embolism as part of chest pain workup. The result of CTA was negative for pulmonary embolism. The patient was diagnosed with acute bronchitis and the patient's symptoms of cough and chest pain were resolved after antibiotic treatment of acute bronchitis. On the images of CTA, a 2.0 × 1.7 cm right paratracheal mediastinal mass was noted which appeared slightly hyperdense or showed mild contrast enhancement (Figure 1).

**Figure 2:**
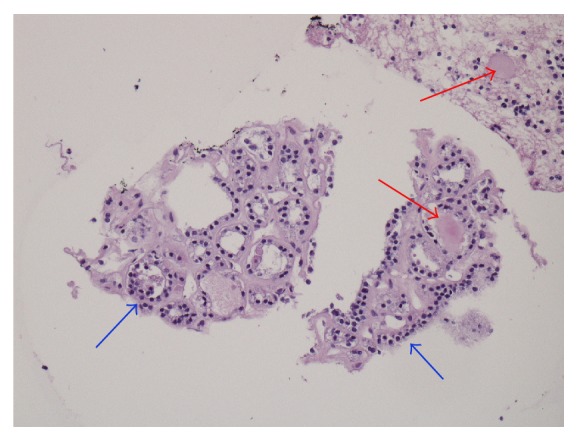
Histological image of ectopic thyroid tissue in mediastinum. Fragments of benign thyroid tissue identified in the cell block prepared from the transbronchial needle aspiration. Bland, uniform thyroid epithelial cells are disposed in the follicular units characteristic of benign thyroid tissue (blue arrow), with focal presence of intrafollicular colloid (red arrow). There is mild perifollicular hyalinization (Hematoxylin and Eosin, 20x).

Differential diagnosis for this upper mediastinal mass included an enlarged lymph node reactive to infection or a chronic inflammatory process, sarcoidosis, or nodal metastasis from occult malignancy. The patient underwent an endobronchial ultrasound-guided biopsy of the right paratracheal mass, which was found to be benign ectopic thyroid tissues by histological analysis of the biopsied tissue samples (Figure 2).

**Figure 3:**
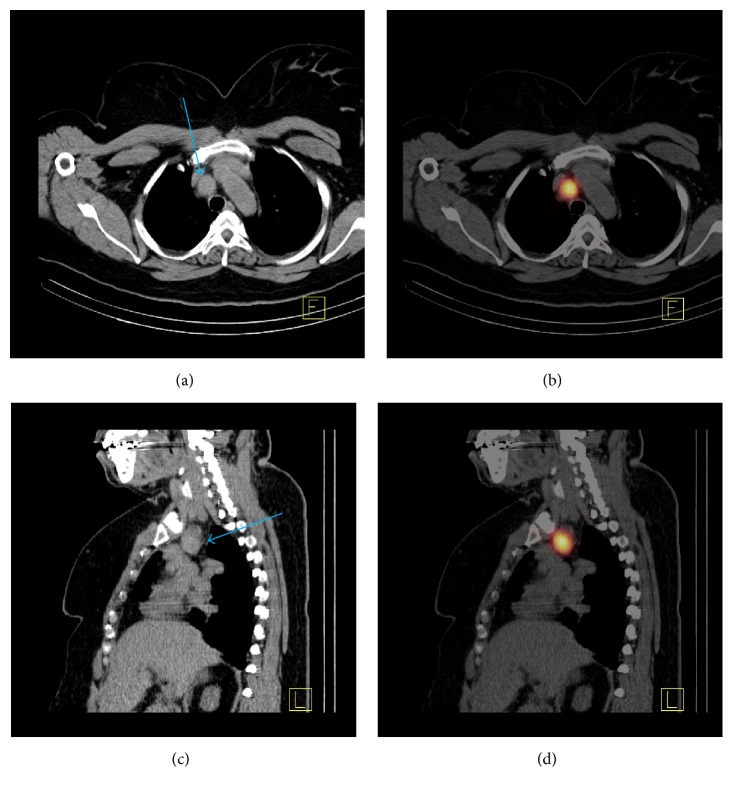
Functional and anatomic imaging of ectopic thyroid tissue in the upper mediastinum by I-123 SPECT/CT. A 2.0 × 1.7 cm right paratracheal mediastinal mass was visualized on the axial (a) and sagittal (c) view images of noncontrast enhanced low dose CT component of I-123 SPECT/CT as indicated by a blue arrow. On the axial (b) and sagittal (d) view images of coregistered I-123 SPECT/CT images, I-123 radioiodine uptake by the right paratracheal mass was visualized as indicated by orange color presentation of I-123 radioiodine activity. The findings of I-123 SPECT/CT further confirmed that the upper mediastinal mass seen on the CTA images represented functional ectopic thyroid tissue, consistent with the results of histological analysis of the tissue samples obtained by endobronchial ultrasound-guided biopsy.

A 24 hours I-123 uptake and scintigraphic scan were performed for further functional characterization of this ectopic thyroid tissue mass within the mediastinum. The 24-hour radioiodine uptake by residual thyroid tissue in the thyroidectomy bed was measured at 1.5% and no thyroid tissue with I-123 uptake was visualized on the surgical bed, compatible with the patient's history of prior total thyroidectomy 10 years ago. One focus of increased I-123 radioiodine accumulation was identified in the region of upper mediastinum on the planar images of I-123 scan. For further anatomic localization of the focal uptake in the upper mediastinum visualized on planar scintigraphic images, a SPECT/CT was performed using a dual headed Siemens Symbia T2 SPECT/CT camera in a method as previously described [22]. On SPECT/CT images, the focus of increased radioiodine uptake in the mediastinum seen on planar imaging was localized to the 2.0 × 1.7 cm right paratracheal mediastinal mass visualized on CTA (Figure 3).

The patient had a history of total thyroidectomy for thyroiditis and nodules 10 years ago. Post-total thyroidectomy hypothyroidism was treated with oral administration of 125 to 137 mg Levoxyl daily for one year. Subsequently, the dose of Levoxyl was reduced to 100 mg/daily and the results of thyroid functional tests were normal with a TSH level of 2.63 mIU/L (normal reference range 0.40–4.50 mIU/L) and a free T4 of 1.6 ng/dL (normal reference range of 0.8–1.8 ng/dL) at 4 years after the patient was maintained on 100 mg of Levoxyl daily for treatment of post-total thyroidectomy hypothyroidism. However, TSH level was low at 0.09 mIU/L and a free T4 level was high at 2.58 ng/dL when a thyroid functional test was performed at 6 days after mediastinal ectopic thyroid tissue was diagnosed with histological analysis of the biopsied tissue samples. In preparation for I-123 SPECT/CT, Levoxyl was stopped for 4 weeks and the patient developed symptoms of hypothyroidism (fatigue, constipation, and hair loss). In view of possible functional activity of benign mediastinal ectopic thyroid tissue confirmed by I-123 SPECT/CT, a reduced dose of 75 mg of Levoxyl daily was prescribed for this patient upon completion of I-123 SPECT/CT. One year later, the results of thyroid functional test were normal with a free T4 level 1.4 ng/dL and a TSH level 2.11 mIU/L. Follow-up CT of chest one year after I-123 SPECT/CT revealed no significant interval changes of the size and morphology of the ectopic thyroid tissue, supporting a conservative management without rebiopsy or surgical resection of the ectopic thyroid tissue within the upper mediastinum.

## 3. Discussion

Ectopic thyroid tissue that coexists with a normally located orthotopic thyroid gland has been reported at equal incidences with ectopic thyroid occurring without a normally located gland. There are rare reports of dual ectopia or two separate foci of ectopic tissue in different locations [23]. Ectopic thyroid tissue should be suspected when a hyperdense mass within mediastinum is detected by CT chest. On CT ectopic thyroid tissue is typically identical in appearance to orthotopic thyroid tissue, a well-circumscribed homogeneous mass with increased attenuation (70 HU ± 10) relative to adjacent skeletal muscle due to iodine content and avidly enhancing on postcontrast images [23, 24]. In the clinical cases of suspected ectopic thyroid tissue, radionuclide imaging with technetium-99m pertechnetate, iodine-123, or iodine-131 is useful for functional assessment of radioiodine uptake by the suspected ectopic thyroid tissues. Retrospectively, it might be desirable to determine whether the hyperdense right paratracheal mass seen on CTA represented an ectopic thyroid tissue mass with I-123 SPECT/CT prior to biopsy. Prebiopsy I-123 SPECT/CT could be used for both functional and anatomic characterization of the right paratracheal mass and guiding tissue biopsy of the mass. Malignancy may occur within ectopic thyroid tissue with a variety of cell types reported. In contrast to ectopic thyroid gland neoplasms, the majority of tumors reported in lingual thyroid tissue are follicular while papillary forms are reported to comprise 23% [2]. Histological analysis of the biopsied tissue samples is desirable to determine benign versus malignancy of ectopic thyroid tissue. In the absence of significant symptoms of hyperthyroidism or mass effect from large ectopic thyroid tissue, management is typically conservative. Ectopic thyroid may be excised when mass effect becomes symptomatic or clinically significant or there is suspicion of malignancy [5, 15].

Majority of patients with ectopic thyroid presented with hypothyroidism which can be medically managed with thyroid hormone supplement. Rare cases of hyperthyroidism with histological features similar to Graves' disease have been reported [17, 18]. The patient of this case report was initially treated with 125 to 137 mg Levoxyl daily after total thyroidectomy. Because the patient's TSH and free T4 levels were not normalized or unstable, the dose of Levoxyl was reduced to 100 mg daily. However, the patient's TSH (0.09 mIU/L) and free T4 levels (2.58 ng/dL) were still abnormal prior to stopping oral administration of Levoxyl (100 mg, daily) in preparation for I-123 SPECT/CT. The results of thyroid functional tests were normal after the dose of Levoxyl was reduced to 75 mg daily after I-123 uptake by the benign mediastinal ectopic thyroid tissue was demonstrated by I-123 SPECT/CT. The results of abnormal thyroid functional tests (low TSH and high free T4) when the patient received 100 to 135 mg of Levoxyl might be related to combined effects of Levoxyl and thyroid hormone produced by the benign ectopic mediastinal thyroid tissues.

In summary, the findings from this case suggested that ectopic thyroid tissue should be suspected for differential diagnosis of incidental finding of hyperdense mediastinal mass on CT. I-123 SPECT/CT can be useful for both functional and anatomic characterization of suspected ectopic thyroid tissue to guide medical management of hypothyroidism, rarely hyperthyroidism, in the patients with ectopic thyroid tissues.
